# Genome-Wide Analysis of Neuroblastomas using High-Density Single Nucleotide Polymorphism Arrays

**DOI:** 10.1371/journal.pone.0000255

**Published:** 2007-02-28

**Authors:** Rani E. George, Edward F. Attiyeh, Shuli Li, Lisa A. Moreau, Donna Neuberg, Cheng Li, Edward A. Fox, Matthew Meyerson, Lisa Diller, Paolo Fortina, A. Thomas Look, John M. Maris

**Affiliations:** 1 Department of Pediatric Oncology, Dana-Farber Cancer Institute, Boston, Massachusetts, United States of America; 2 Division of Oncology, Children's Hospital of Philadelphia, University of Pennsylvania School of Medicine, Philadelphia, Pennsylvania, United States of America; 3 Department of Biostatistics and Computational Biology, Dana-Farber Cancer Institute, Boston, Massachusetts, United States of America; 4 Department of Biostatistics, Harvard School of Public Health, Boston, Massachusetts, United States of America; 5 Microarray Core Facility, Dana-Farber Cancer Institute, Boston, Massachusetts, United States of America; 6 Department of Medical Oncology, Dana-Farber Cancer Institute, Boston, Massachusetts, United States of America; 7 Cardeza Foundation for Hematologic Research, Thomas Jefferson University, Philadelphia, Pennsylvania, United States of America; Sloan-Kettering Cancer Center, United States of America

## Abstract

**Background:**

Neuroblastomas are characterized by chromosomal alterations with biological and clinical significance. We analyzed paired blood and primary tumor samples from 22 children with high-risk neuroblastoma for loss of heterozygosity (LOH) and DNA copy number change using the Affymetrix 10K single nucleotide polymorphism (SNP) array.

**Findings:**

Multiple areas of LOH and copy number gain were seen. The most commonly observed area of LOH was on chromosome arm 11q (15/22 samples; 68%). Chromosome 11q LOH was highly associated with occurrence of chromosome 3p LOH: 9 of the 15 samples with 11q LOH had concomitant 3p LOH (P = 0.016). Chromosome 1p LOH was seen in one-third of cases. LOH events on chromosomes 11q and 1p were generally accompanied by copy number loss, indicating hemizygous deletion within these regions. The one exception was on chromosome 11p, where LOH in all four cases was accompanied by normal copy number or diploidy, implying uniparental disomy. Gain of copy number was most frequently observed on chromosome arm 17q (21/22 samples; 95%) and was associated with allelic imbalance in six samples. Amplification of MYCN was also noted, and also amplification of a second gene, ALK, in a single case.

**Conclusions:**

This analysis demonstrates the power of SNP arrays for high-resolution determination of LOH and DNA copy number change in neuroblastoma, a tumor in which specific allelic changes drive clinical outcome and selection of therapy.

## Introduction

Neuroblastoma, like most human cancers, is characterized by genomic instability, manifested at the chromosomal level as allelic gain, loss, or rearrangement. These aberrations frequently are associated with a particular disease phenotype, such as widespread metastasis or early relapse, and are therefore clinically important. Allelic imbalance in solid tumors can be detected by a variety of methods, including sequence-specific hybridization of tumor cell DNA at polymorphic loci across the human genome. Single nucleotide polymorphisms (SNPs) provide a high-density method for large-scale analyses of polymorphic markers. SNP arrays provide a feasible means of conducting high-throughput, genome-wide screens for allelic imbalance [1]. SNP array analysis enables detection of genotypic alterations in the tumors of individual patients and, in principle, identification of new areas with common allelic imbalance that could harbor potential tumor suppressors. Additionally, this method allows for simultaneous measurement of DNA copy number [2]. SNP array analysis shows high concordance with microsatellite methods, but allows detection of smaller regions of LOH that maybe missed by microsatellite mapping [3]. In addition, because LOH can occur without change in DNA copy number, SNP arrays offer more potential than comparative genomic hybridization in detecting such events. This type of analysis has been used successfully to assess different cancers [4]–[6].

Studies of neuroblastoma cells consistently identify LOH at several chromosomal regions, some of which correlate with a poor outcome, but tumor suppressor genes in these regions remain to be identified. An example is the deletion of chromosome band 1p36, which is strongly correlated with *MYCN* gene amplification, a very poor prognostic marker in neuroblastoma [7], [8], and which by itself is also commonly associated with an advanced disease stage and a poor outcome [9]. Chromosome band 11q23 LOH is inversely related to *MYCN* amplification but also reliably identifies patients at high risk for disease relapse [10]. Additionally, unbalanced gain of chromosome 17q is associated with high-risk disease features, such as advanced disease stage and age at diagnosis, *MYCN* amplification, and 1p36 LOH, and a decreased survival probability [11]. Thus, the available data clearly indicate that DNA copy number aberrations are significant predictors of disease phenotype and clinical behavior in neuroblastoma, including likelihood of response to chemotherapy and/or eventual disease relapse. A chip-based method for whole-genome evaluation of DNA alterations has the potential to streamline gene discovery efforts and provide genomic signatures that may be useful in predicting prognosis.

## Results

### Characteristics of the patients and tumor samples

Age at diagnosis ranged from 6.5 months to 11.6 years, with a median of 3.3 years. Majority (19/22 or 86.4%) of patients were over a year old. All but one patient had metastatic disease at diagnosis. *MYCN* amplification occurred in 5 tumors (22.7%) and hyperdiploid DNA content in 15/22 (68.2%) tumors. Majority (20/22) of the tumors had unfavorable Shimada histology. All 22 patients met the Children's Oncology Group (COG) criteria for having high-risk disease [12].

### SNP array analysis

**Figure 1:**
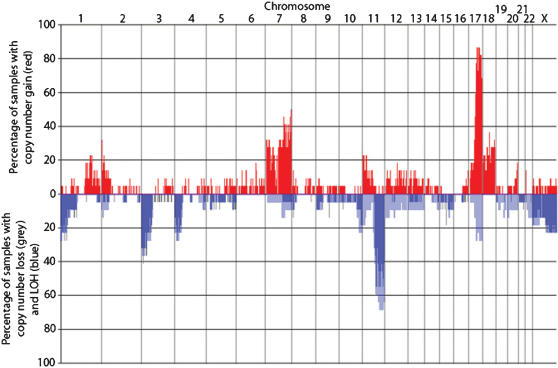
Frequency of chromosomal aberrations. Fraction of samples with copy number >2.8 (red bars above baseline), copy number <1.2 (grey bars below baseline), and LOH (blue bars below baseline). SNPs are mapped according to their chromosomal position, from chromosome 1 on the left to chromosome X on the right. (Copy number alterations for chromosome X in samples derived from males were counted if copy number was >1.4 or <0.6.)

The average genotype call rates for tumor and normal samples were 93.7±3.7% (range: 82.4–98.5%) and 96.1±4.0% (range: 85.4–99.0%), respectively. On average, 29.3% of the SNP loci were informative (heterozygous in normal samples and all SNP calls made in tumor samples except No call), with a range of 20.6–35.3%. All samples assayed showed DNA copy number aberrations and LOH (Figure 1). The median percentage of the genome with copy number alterations in our sample set was 8.4% (range: 0.6–15.1%).

### Detection of LOH

**Figure 2:**
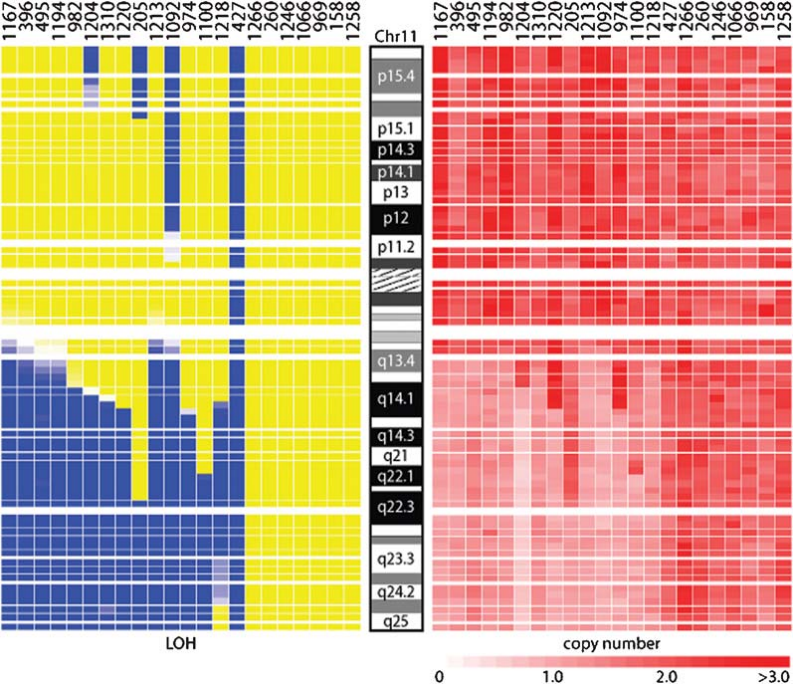
LOH and copy number changes on chromosome 11. SNP array analysis of neuroblastoma tumor samples and matched constitutional DNA showing LOH on the left and copy number on the right of the chromosome 11 ideogram. For each sample, chromosome 11 is depicted as a vertical bar in both the LOH and copy number panels. Blue areas represent regions of LOH, while yellow denotes retention of heterozygosity. Copy number is marked by shades of red, with ≤1 copy in light red and ≥3 copies in dark red (see scale at the bottom of the panel). Both chromosome 11q and 11p LOH, as well as gain of 11p, are shown.

**Figure 3:**
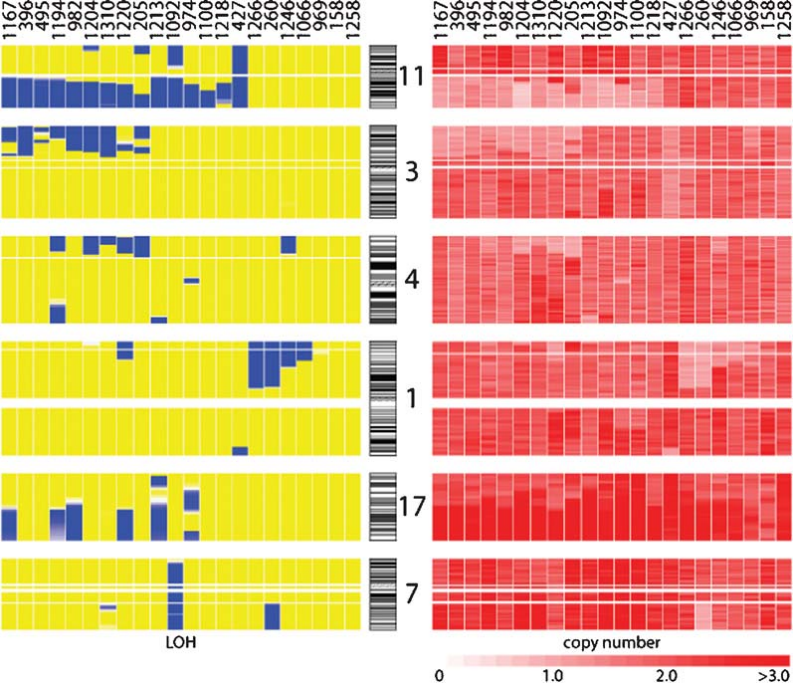
LOH and copy number changes on chromosomes 11, 3, 4, 1, 17, and 7. Global view of common areas of LOH (left) and copy number change (right) in 22 primary neuroblastomas. Each sample is depicted as a series of vertical bars in both the LOH and copy number panels. Blue areas represent regions of LOH, while yellow denotes retention of heterozygosity. Copy number is marked by shades of red, with ≤1 copy in light red and ≥3 copies in dark red (see scale at the bottom of the panel).

Six main regions of LOH were identified in this analysis (Table 1). The most frequent region, present in 15 of 22 tumor samples (68%; Figure 2), was on chromosome arm 11q. All 15 cases showed large regions of LOH with retention of heterozygosity of portions of 11p and 11q in all but one sample which demonstrated whole chromosome 11 LOH (#427). A total of 6/14 cases (43%) with 11q copy number loss showed a concomitant gain in copy number on 11p. LOH of chromosome 3p was found in 45% of cases (Table 1). Chromosome arm 11q and 3p LOH tended to occur together: 9 of 15 cases with 11q deletion also had loss of 3p (P = 0.016) (Figure 3). Only one case with 3p LOH lacked an associated 11q LOH. Other regions of the genome that showed appreciable LOH were 1p and 4p, which were detected in approximately 32% and 27% of cases, respectively (Table 1). LOH of chromosome 11p was also observed in 4 samples, and chromosome 14q LOH observed in 2 samples (Figure 2).

**Table 1:** Common regions of LOH in high-risk neuroblastoma and corresponding copy number change (A), and areas of copy number gain (B) identified by 10K SNP array analysis

A. Areas of LOH					
Chromosomal Region	Smallest Region of Overlap (Mb)	N (%)		Corresponding Copy Number Change (N, %)	Suggested Mechanism
1p	ter-38.9	7 (32%)		Reduction (7, 100%)	Hemizygous deletion
3p	ter-44.2	10 (45%)		Reduction (10, 100%)	Hemizygous deletion
4p	ter-23.2	6 (27%)		Reduction (6, 100%)	Hemizygous deletion
11q	105.6-134.5	15 (68%)		Reduction (14, 93%)	Hemizygous deletion
				No change (1, 7%)	Copy neutral events
11p	ter-15.2	4 (18%)		No change (4, 100%)	Copy neutral events
14q	98.9-106.3	2 (9%)		Reduction (2, 100%)	Hemizygous deletion
**B. Areas of Copy Number Gain**					
**Chromosomal Region**			**Smallest Region of Overlap (Mb)**		**Tumors with Copy Number Gain (%)**
1q			183.65–197.3		12 (55%)
			222.05–228.6		
2p			7–29.6		11 (50%)
7					15 (68%)
7p only			ter–34.75		2 (9%)
7q only			107.02-ter		3 (14%)
Whole chromosome 7					10 (45%)
11p			16.1–26.0		6 (27%)
17q					21 (95%)
17q only			44.83-ter		16 (73%)
Whole chromosome 17					5 (23%)
18q					11 (50%)
18q only			41.76-ter		6 (27%)
Whole chromosome 18					5 (23%)

### Mechanisms of LOH

Almost all of the regions of LOH resulted from copy number loss. In the case of chromosome 11q LOH, all 14 samples with apparent chromosome 11 breakpoints showed copy number reduction of the region of LOH, while the one sample with whole chromosome 11 LOH maintained two allelic copies of the entire chromosome (Figure 2). Copy number loss accompanied LOH of chromosomes 3p, 1p, 4p and 14q. The 4 samples with chromosome 11p LOH, however, had diploid copy numbers. In addition, in 6 tumors with chromosome 17q LOH, there was a gain in copy number. In one case (#427) an apparent copy-number reduction at chromosome 3p was not associated with LOH (Figure 3).

Although no homozygous deletions were unequivocally identified in this dataset, there was a potential homozygous deletion, defined as an inferred copy number of <0.3 in at least 2 consecutive SNPs. The flanking SNPs define a 124 kb area at 10q26.2, to which no known genes have been mapped.

### Detection of copy number gain

**Figure 4:**
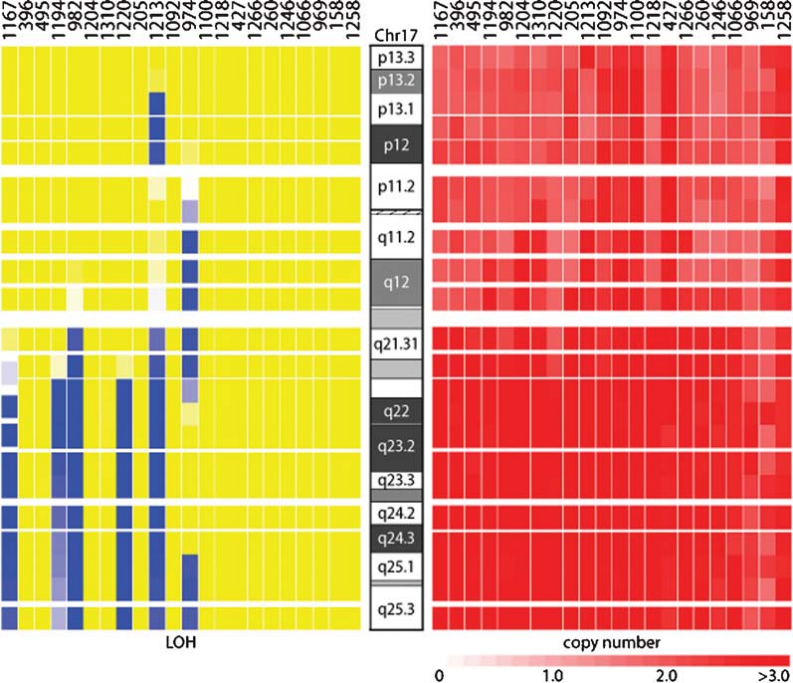
Copy number and LOH analysis of chromosome 17. SNP array analysis of neuroblastoma tumor samples and matched constitutional DNA showing LOH on the left and copy number on the right of the chromosome 17 ideogram. For each sample, chromosome 17 is depicted as a vertical bar in both the LOH and copy number panels. Blue areas represent regions of LOH, while yellow denotes retention of heterozygosity. Copy number is marked by shades of red, with ≤1 copy in light red and ≥3 copies in dark red (see scale at the bottom of the panel). This figure depicts copy number gain of chromosome 17q in 21 out of 22 samples.

Six chromosomal regions showed frequent gains of copy number (Table 1; Fig. 1). The most common of these was chromosome arm 17q in 21 of the samples (Figure 4). Each of these 21 samples had 3 to 4 copies of 17q; 16 had an unbalanced gain of 17q without a gain of 17p. Of the 21 tumors with a gain of 17q, 6 also showed LOH on 17q (Figure 4).

A gain of chromosome 1q was found in 55% of the tumors, while gain of chromosome arm 2p, the site of the *MYCN* oncogene, was detected in 50% of the cases. Other notable findings were whole chromosome 7 gain in 10 samples (45%), gain of whole chromosome 18 in 5 samples, and isolated chromosome 18q gain in 6 samples.

### Regions of chromosomal amplification

**Figure 5:**
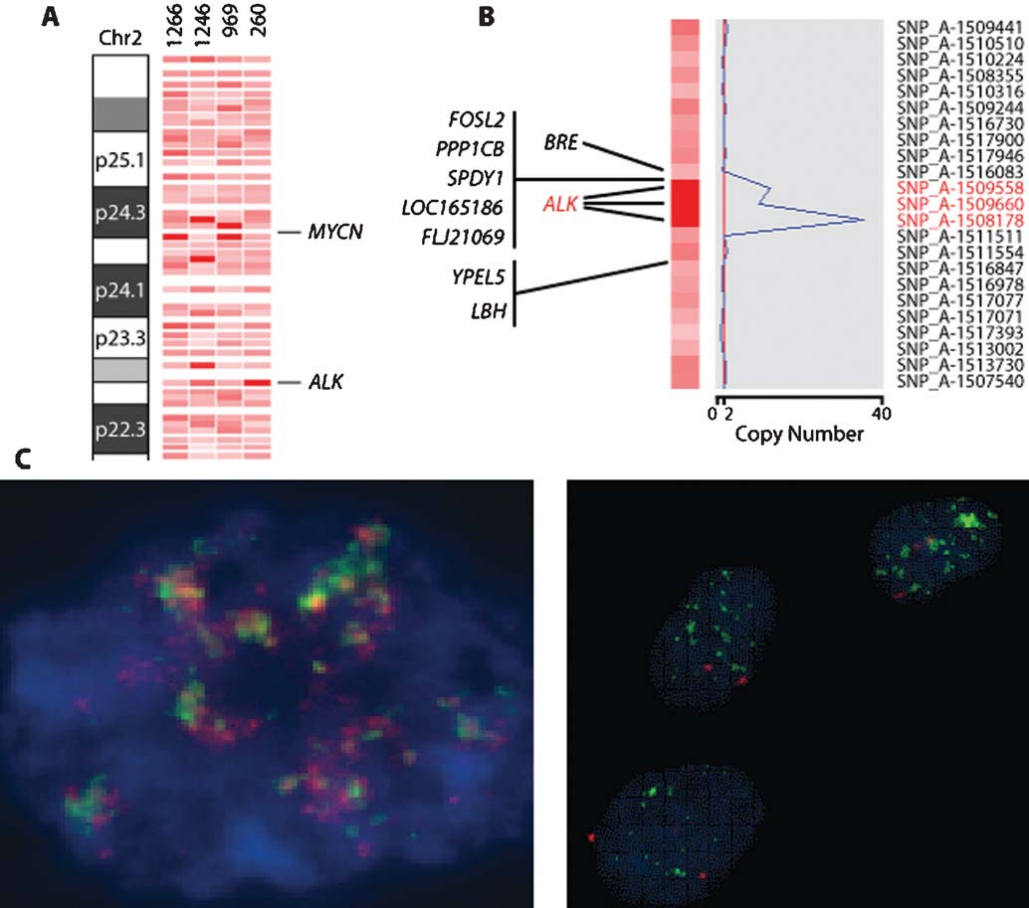
Amplifications on chromosome 2p. (A) SNP copy number analysis of a portion of chromosome arm 2p demonstrating amplification (represented by the darker red SNPs) at the *MYCN* gene locus (2p24.3) in three tumor samples and at the *ALK* gene locus (2p23.2) in one sample. (B) Individual SNP copy number assessment of the SNPs surrounding the *ALK* locus. The blue curve in the graph on the right indicates the degree of amplification of each SNP from 0 on the left to 40 on the right. The red vertical line indicates a copy number of 2. (C) FISH of neuroblastoma with the *MYCN* probe showing multiple copies of *MYCN* (left panel), and FISH of the same tumor using the *ALK* break apart probe showing amplification of *ALK* (right panel).

High-level gene amplification events were less frequent than LOH or copy number gains. *MYCN* amplification was seen in 3 cases on 10K SNP array analysis. However, 10K array analysis did not detect *MYCN* amplification in 2 samples that showed multiple copies by FISH. These two samples were re-analyzed using the higher density 250K SNP array and clearly showed *MYCN* amplification. A second area of amplification was found in one tumor at chromosome 2p23, the site of the anaplastic lymphoma kinase (*ALK*) gene on the 10K array. Amplification was observed in three consecutive SNPs (20 copies of each SNP) encompassing the *ALK* gene (Figure 5). Although both *MYCN* and *ALK* are located on chromosome 2, at 2p24.1, and 2p23.2 respectively, there were more than 21 unamplified SNPs between the two genes.

### Deletion mapping

Overlapping areas of deletion defined the smallest regions of overlap (SRO). All 11q deletions extended to the telomere; the smallest covered 29.8 Mb from 11q22.3-tel. There were two common regions of deletion on 3p: a 28.4 Mb region within 3p26.3-24.1 and a 12.6 Mb region within 3p21.31-14.3.

### Comparison with other methods of detecting allelic imbalance

10K SNP array analysis did not detect *MYCN* amplification in 2 samples that showed multiple copies by FISH (concordant results in 3/5 samples). However, *MYCN* amplification was clearly seen when these two tumors were analyzed by the higher density 250K StyI array (Affymetrix Inc.). Non-amplified *MYCN* was confirmed by both FISH and 10K SNP array analysis in 17 samples. One sample scored as LOH on chromosome 1p by PCR genotyping but did not display LOH by SNP. Although all expected samples with 11q LOH were detected by both methods, 4 samples previously determined to have whole chromosome 11 LOH by microsatellite mapping actually had areas of chromosome 11 without LOH on SNP array analysis. The SNP array analysis was concordant in 13/16 cases of unbalanced 17q gain by PCR.

### Associations between allelic imbalances and *MYCN* amplification

Although the number of samples was small, chromosome arm 1p LOH was significantly correlated with *MYCN* amplification (P = 0.0002). On the other hand, 11q LOH was significantly associated with non-amplified *MYCN* (P = 0.0008), as was whole chromosome 7 gain (P = 0.04).

## Discussion

This study represents the application of SNP arrays in the genome-wide analysis of LOH and DNA copy number change in neuroblastoma. We have previously reported a method of assessing region-specific LOH in neuroblastoma using a SNP-based tag-array platform [13]. In this assay, informative SNPs from nine known regions of LOH in neuroblastoma were identified and used in designing a customizable platform of 500 SNPs. This array reliably detected LOH in 6 or more genomic regions, obviated the requirement for matched constitutional DNA, and would be very useful as a neuroblastoma-specific chip to achieve prognostication, but would fail to uncover novel areas of LOH and copy number gain. The Affymetrix 10K array we used in this study permits the simultaneous genotyping of 11,560 SNPs spaced throughout the human genome at a median intermarker distance of 100 kb. SNP array analysis identified multiple known areas of allelic imbalance in neuroblastomas, such as LOH of chromosomes 11q, 3p, 1p, and 4p. In addition, novel findings were the uniparental disomy that accompanied chromosome 11p LOH and the finding of allelic imbalance with chromosome 17q gain. Amplification of *ALK*, a gene with transforming activity in anaplastic large cell lymphoma was also seen.

Deletions of 11q have been noted in approximately 35% to 45% of neuroblastomas using microsatellite markers, CGH, and FISH and are consistently seen in *MYCN* non-amplified tumors [10], [14], [15]. None of the tumors in our study with 11q LOH had *MYCN* amplification [10]. Moreover, 9 of 10 tumors with 3p LOH had concomitant 11q LOH, as previously reported [15]. The nonrandom association of 3p LOH with 11q LOH in neuroblastoma likely signifies losses of tumor suppressor genes on these chromosome arms and may represent a synergistic alternative oncogenic pathway in high-risk tumors lacking *MYCN* amplification. Chromosome 1p LOH was documented in 32% of samples in this study and was associated with *MYCN* amplification, as described previously [9], [10]. The deletions of 1p defined an SRO of 7.8 Mb within 1p36.32-36.22, which includes the common region of deletion defined by White et al [16].

SNP array analysis simultaneously detects LOH and copy number changes and therefore can be used to elucidate the mechanisms underlying LOH. In this analysis, LOH was accompanied by copy number loss, normal copy number, and copy number gain. Copy number loss accounted for most of the regions of LOH and was found on chromosomes 11q, 3p, 1p, and 4p, in which genes on the retained alleles are thought to be haploinsufficient or inactivated by intrachromosomal deletions, mutations, or epigenetic phenomena.

LOH with a normal copy number was documented in all 4 of the tumors with LOH on chromosome 11p. Such LOH without accompanying copy number reduction, termed “copy-neutral” events have been reported in rhabdomyosarcoma [17], breast cancer [18], and acute myeloid leukemia [19], and are thought to be due to uniparental disomy, which result in the loss of one allele and duplication of the retained allele. The fact that in 3 tumors (#1204, 205, and 1092) the regions of 11p LOH extended to the telomere suggests that this phenomenon is likely due to somatic recombination. In tumor #427, entire chromosome 11 LOH is seen, suggesting either a somatic recombination event close to the centromere or a nondisjunction event with subsequent chromosomal duplication. This may result in gain of function of the genes in this region, as for example, *IGF2*, which is known to induce neuroblastoma cell proliferation [20]. Alternatively, the targeted gene within this region on the duplicated allele may also contain inactivating mutations or be suppressed by epigenetic mechanisms, resulting in loss of function. Further studies are required to determine which gene or genes are mutated and how the mutations that led to their inactivation were selected for in the first place.

We also observed LOH accompanied by an increase in copy number on chromosome 17q. The loss of one allele followed by a gain of the remaining allele would suggest the need to eliminate the wild-type function of an activated oncogene. This phenomenon has been suggested previously in osteosarcomas, where LOH was accompanied by a significant increase in copy number [21]. Alternatively, copy number gain and the resulting allelic imbalance may result in a false reading of LOH by the genotyping algorithm [22]. Indeed, all three of our samples with *MYCN* amplification on chromosome 2p24 appeared to have LOH at that locus. Further studies will be needed to determine whether chromosome 17q LOH in fact accompanies 17q gain in some cases of neuroblastoma or whether this phenomenon is merely an indication of allelic imbalance.

The association of gain of chromosome arm 11p with 11q loss was seen in this analysis and has been reported in up to 55% of neuroblastomas with 11q loss [23], suggesting cooperation between tumor suppressors and oncogenes within these regions. Chromosome 17q gain was observed in all but 1 of the 22 (95%) tumors, and thus was the most prevalent abnormality in our series [11], [24]. Gain of either the entire chromosome 7 or its long arm has been detected in 40% of neuroblastomas by CGH [23]; in our study, 7q gain was documented in 10/22 (45%) of tumors. In addition, chromosome 7q gain has been significantly correlated with lack of *MYCN* amplification, which was also the case in our series. Chromosome 7q gain has also been shown to occur through an unbalanced t(3;7) translocation with loss of 3p material [23]. In our series, 50% (7/14) of tumor samples with 7q gain had concomitant loss of 3p, suggesting synergistic tumor suppressor pathways.

We identified another area of amplification in our series, at chromosome 2p23, the locus of the *ALK* gene [25]. *ALK* encodes a tyrosine kinase receptor and was first identified as a component of the *NPM-ALK* fusion gene in anaplastic large cell lymphoma. It was shown to be constitutively activated by amplification at the *ALK* locus in neuroblastoma cell lines [26], and suppression of activated *ALK* in such cells induced apoptotic changes through reduction in the phosphorylation of ShcC, a Src homology 2 domain-containing adaptor molecule involved in signaling through the MAP kinase pathway. Although *ALK* is commonly expressed in the cytoplasm of neuroblastoma cells, its amplification has so far been demonstrated in only 1 of 85 tumor samples [27]. Amplification is correlated with expression. Although the tumor sample with *ALK* amplification was known to harbor *MYCN* amplification by FISH, *MYCN* amplification was not detected on the 10K array due to the small *MYCN* amplicon size. When this same sample was analyzed on a 250K array, *MYCN* amplification was noted. On this latter array there were several intervening SNPs between the *MYCN* and *ALK* genes that did not show amplification, however this does not rule out *ALK* coamplification with *MYCN*, as discontinuous regions of amplification may have occurred. Nevertheless, the finding of *ALK* amplification in neuroblastoma may provide a novel therapeutic target that could be tested using available *ALK* inhibitor compounds.

SNP arrays have many advantages over more conventional methods of cancer genome analysis in terms of efficiency, precision and minimal DNA requirements, and may well become the dominant technology for performing genome-wide tumor cell LOH and copy number measurements. This application seems especially relevant to large studies such as those of the Children's Oncology Group, in which treatment for neuroblastoma patients is already based on genetic abnormalities and further stratifications are planned based on 1p and 11q LOH [10]. Although the use of high-density SNP arrays to provide clinically relevant information has great appeal, this strategy must first be validated using newer generation SNP arrays containing probes for more SNP markers with higher informative rates. Ultimately, the results of cancer genome-wide allelotyping by SNP array analysis may predict responses to specific therapies, allowing more efficient modification of regimens for individual patients.

## Methods

### Patients and tumor samples

Samples were identified from the Children's Oncology Group (COG) Neuroblastoma Nucleic Acids Bank with the only inclusion criteria being 1) availability of matched constitutional DNA from peripheral blood mononuclear cells; 2) samples obtained at original diagnosis and immediately snap frozen; and 3) a tumor cell content of more than 90% based on differential count, clonal hyperdiploid percentage in some tumors, and direct examination of H&E-stained tumors in a subset of cases. Patients were staged according to the International Neuroblastoma Staging System [28] and histology was analyzed by the Shimada Pathology Classification [29]. *MYCN* gene amplification and DNA ploidy were determined as previously described [30], [31]. LOH and chromosome gain status were determined on chromosome arms 1p, 3p, 11q, and 17q using conventional microsatellite markers as previously described [10]. This project received joint approval from the Institutional Review Boards of the Dana-Farber Cancer Institute and the Children's Hospital of Philadelphia.

### Preparation of DNA target and SNP genotyping

The Affymetrix 10K SNP array was used in all initial experiments and the 250K array in subsequent ones, according to the methods described by the manufacturer. Briefly, 250 ng of tumor and control DNA were digested with the appropriate restriction enzyme (*Xba1* for the 10K array and *Sty1* for the 250K array) in parallel reactions, and ligated to a universal adaptor sequence. For each sample, the ligated DNA was PCR amplified under recommended conditions, using primers complementary to the universal adapters. The PCR products were then fragmented and end-labeled with biotinylated ddATP using terminal deoxynucleotidyl transferase. The labeled DNA was then hybridized to the 10K SNP chips, washed, incubated with streptavidin, and stained with biotinylated antistreptavidin antibody and a streptavidin R-phycoerythrin conjugate. The chips were scanned with an HP scanner (Hewlett-Packard, Palo Alto, CA) according to the manufacturer's recommendations. Affymetrix genotyping software (Affymetrix GeneChip 5.0) was used to examine the SNP hybridization patterns and to make SNP calls of all loci in each of the tumor samples and their corresponding matched controls.

### Analysis of LOH

The resulting data were analyzed with the dChip software package [32], which is freely available at www.dchip.org. At each locus, 1 of 5 LOH calls was assigned by the dChip algorithm in a pair of normal and tumor cells, according to the following rules: allelic imbalance or LOH (AB in normal, A or B in tumor; blue); retention of heterozygosity (AB in both normal and tumor; yellow,); non-informative (A or B in normal, gray); no call (SNP No Call in normal or tumor sample, white), or conflict (A or B in normal and AB in tumor, red) (Fig. 2). A “region of LOH” was assigned when there were at least 2 consecutive SNPs showing LOH. The overall call rate was calculated as the number of SNPs assigned A, B, or AB (all SNP calls except No call) divided by the total number of SNPs in the array.

### Measurement of DNA copy number change

Copy number change was measured based on comparing the hybridization intensity between normal and tumor samples using the dChip software, with a sliding window of 3 SNPs as previously described [2]. Copy number gain was defined as between 2.8 and 5 copies (alleles). Amplification was defined as an inferred copy number of greater than 5 in 3 consecutive SNPs. Copy number loss was defined as less than 1.2 copies. Homozygous loss was defined as an inferred copy number of <0.3 in at least 2 consecutive SNPs.

### Comparison with other methods of detecting allelic imbalance

Results of the SNP arrays were compared with those of conventional methods of detecting genomic aberrations in cancer cells, including fluorescence *in situ* hybridization (FISH) for *MYCN* amplification as described previously [30], microsatellite genotyping for LOH at 1p36 and 11q23 [10], and quantitative real-time PCR for unbalanced 17q gain [33].

### Statistical analysis

Tests of association between chromosomal abnormalities and *MYCN* amplification were performed using a two-sided Fisher's Exact test.
